# Busting the myth of spontaneous formation of H_2_O_2_ at the air–water interface: contributions of the liquid–solid interface and dissolved oxygen exposed†

**DOI:** 10.1039/d3sc06534k

**Published:** 2024-01-24

**Authors:** Muzzamil Ahmad Eatoo, Himanshu Mishra

**Affiliations:** a Environmental Science and Engineering (EnSE) Program, Biological and Environmental Science and Engineering (BESE) Division, King Abdullah University of Science and Technology (KAUST) 23955-6900 Thuwal Kingdom of Saudi Arabia; b Water Desalination and Reuse Center (WDRC), King Abdullah University of Science and Technology (KAUST) 23955-6900 Thuwal Kingdom of Saudi Arabia Himanshu.Mishra@kaust.edu.sa; c Center for Desert Agriculture (CDA), King Abdullah University of Science and Technology (KAUST) 23955-6900 Thuwal Kingdom of Saudi Arabia

## Abstract

Recent reports on the spontaneous formation of hydrogen peroxide (H_2_O_2_) at the air–water and solid–water interfaces challenge our current understanding of aquatic chemistry and have ramifications on atmosphere chemistry models, surface science, and green chemistry. Suggested mechanisms underlying this chemical transformation include ultrahigh instantaneous electric fields at the air–water interface and the oxidation of water and reduction of the solid at the solid–water interface. Here, we revisit this curious problem with NMR spectroscopy (with an H_2_O_2_ detection limit ≥50 nM) and pay special attention to the effects of nebulizing gas, dissolved oxygen content, and the solid–water interface on this chemical transformation in condensed and sprayed water microdroplets. Experiments reveal that the reduction of dissolved oxygen at the solid–water interface predominantly contributes to the H_2_O_2_ formation (not the oxidation of hydroxyl ions at the air–water interface or the oxidation of water at the solid–water interface). We find that the H_2_O_2_ formation is accompanied by the consumption (*i.e.*, reduction) of dissolved oxygen and the oxidation of the solid surface, *i.e.*, in the absence of dissolved oxygen, the formation of H_2_O_2_(aq) is not observed within the detection limit of ≥50 nM. Remarkably, the tendency of the solids investigated in this work towards forming H_2_O_2_ in water followed the same order as their positions in the classic Galvanic series. These findings bust the prevailing myths surrounding H_2_O_2_ formation due to the air–water interface, the ultrahigh electric fields therein, or the micro-scale of droplets. The hitherto unrealized role of the oxidation of the solid surface due to dissolved oxygen in the formation of H_2_O_2_ is exposed. These findings are especially relevant to corrosion science, surface science, and electrochemistry, among others.

## Abstract 2

The air–water interface is not the site for H_2_O_2_(aq) formation; instead, it takes place at the solid–water interface.

## Graphical abstract

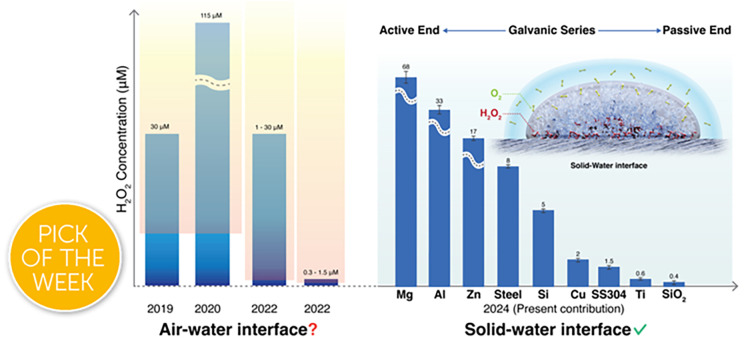

## Introduction

Hydrogen peroxide (H_2_O_2_) is an industrial chemical with a wide range of applications, such as disinfection,^1^ chemical synthesis,^2^ rocket propulsion,^3^ and wastewater treatment.^4^ The current production of H_2_O_2_ at scale relies on the anthraquinone cycling process, requiring significant energy and generating waste,^5^ necessitating sustainable alternatives. Recent reports on the laboratory-scale production of H_2_O_2_*via* electrochemical oxygen reduction are promising.^6–8^ However, electrochemical methods are not devoid of shortcomings; the process is complex and has the risk of spontaneous combustion and explosion. Multiple side reactions may further restrict the scalability of the process.

In this context, sensational reports on the spontaneous formation of H_2_O_2_ at the aerial interface of water microdroplets seem enticing.^9–13^ Specifically, about 30 μM H_2_O_2_ was found in water microdroplets with a diameter of ≤20 μm sprayed *via* pressurized gas,^9^ and ≤115 μM H_2_O_2_ was discovered in condensed water microdroplets on common substrates in the relative humidity range of 40% to 70%.^10^ The presence of ultrahigh electric fields on microdroplet surfaces has been speculated to be the underlying cause.^9,10^ Moreover, studies have noted the implications for atmospheric chemistry,^14^ human health and bactericidal applications,^13^ green chemistry,^9,14^ and the seasonality of diseases due to the Goldilocks effect.^12,15^ We introduce the investigation of this chemical transformation by noting that the examination of water's interfaces is notorious for artifacts arising from contamination, incorrect interpretations of experiments, and the lack of encompassing multiscale computational models.^16–38^ Experience in microdroplet chemistry has demonstrated the need to stress-test the conclusion *via* multiple experimental techniques.

The prospect of aerosolized water microdroplets producing H_2_O_2_ is appealing due to its greenness and potential ease of application.^13,39^ While some experiments,^40–43^ computer simulations,^44,45^ and a Gedankenexperiment^46^ have given credence to the claim of spontaneous H_2_O_2_ formation at the air–water interface, others have disagreed.^47–54^ We commenced the investigation in 2019 by comparing the various commercially available assays for H_2_O_2_(aq). Compared to the potassium titanium oxalate assay (PTO; detection limit ≥10 μM), used in the original reports,^9,10^ the hydrogen peroxide assay kit (HPAK) affords a 40-times lower detection limit (≥250 nM). Equipped with HPAK, we employed a glovebox to assess H_2_O_2_(aq) concentrations in condensed water microdroplets generated in an N_2_(g) environment by gently heating water (50–70 °C). This experiment revealed that the H_2_O_2_ concentrations in the bulk water and the condensates were indistinguishable.^47^ We also discovered that if the condensates were produced *via* ultrasonic humidifiers, about 1 μM H_2_O_2_(aq) was produced in the water reservoir, the mist, and the condensates.^47^ This result was rationalized on the basis of the cavitating bubbles formed under ultrasonic acoustic pressure in bulk water, which is known to produce OH˙ radicals.^49,55–58^ However, why Zare & co-workers found ≤115 μM H_2_O_2_ in their experiments remained unclear.

From 2020 to 2021, we broadened the investigation to include microdroplets produced by pneumatic sprays. This device, like the one in the original report,^9^ facilitated the gas flow speeds of 100 to 1000 ms^−1^, breaking up water droplets to form sprays. In all these studies, (i) sprayed water microdroplets were collected in glass bottles, and (ii) condensed microdroplets were formed on SiO_2_/Si wafers. We discovered that the ppm-level of spontaneous H_2_O_2_ formation (1 ppm = 29.4 μM H_2_O_2_) occurred only in the presence of ozone (O_3_(g)).^48^

In their latest report, Zare & co-workers^59^ repeated the spray experiments in a controlled (ozone-free) gas environment. They employed ^1^H-NMR to quantify H_2_O_2_(aq) at a 40 nM resolution following the protocol of Bax *et al.* (Bruker 600 MHz Avance III, noncryogenic probe, 20 000 scans with 0.1 s acquisition time).^41,60^ When the water was injected through copper tubing in the flow rate range of 25 to 150 μL min^−1^*via* a pressurized N_2_ at 100 psi (6.8 atm), the H_2_O_2_(aq) concentration in the sprays ranged from 1.5 to 0.3 μM (95–99% reduction from the original report^9^). They also found that, for a fixed liquid flow rate, as the nebulizing gas (fixed line pressure) was changed from (i) N_2_ to (ii) N_2_ + O_2_ (2%) to (iii) N_2_ + O_2_ (21%) to (iv) O_2_ (100%), the H_2_O_2_(aq) concentration increased from (i) 0.49 ± 0.05 μM to (ii) 0.69 ± 0.05 μM to (iii) 1.12 ± 0.02 μM to (iv) 2.00 ± 0.05 μM, respectively.^59^ Based on these observations, they contend that their original claims were correct (*i.e.*, the microdroplet air–water interface spontaneously produces H_2_O_2_).

Even if we assume that the latest claim is valid, the previous reports,^9,10^ which applied the PTO assay (H_2_O_2_ detection limit ≥10 μM), could not have detected the 0.30 to 2.00 μM H_2_O_2_(aq) concentrations (*i.e.*, they were reporting artifacts of the ambient ozone gas^48^). This admission may help explain the latest^59^ and previous^9^ reports by Zare & co-workers of contradictory trends in the H_2_O_2_(aq) concentrations in water microdroplets when the concentration of dissolved oxygen (O_2_(aq)) is increased (explained in concluding remarks).

Lastly, two new experimental reports have surfaced in which H_2_O_2_ formation is also observed at the silica–water interface when (i) liquid water passes through a polydimethylsiloxane microfluidic chip placed on glass,^11^ and (ii) water vapor passes through a packed bed of SiO_2_ nanoparticles.^15^ Regarding the mechanism, the authors stated, “In fact, our proposed mechanism is built around the hypothesis that the overlap between the electron clouds of the water molecule and the solid surface during the contact will lead to the generation of H_2_O^+^ and OH˙”.^11^ They also presented an example: “Then, the electron may transfer from the water molecule to the surface of SiO_2_, which is the so-called contact electrification”.^15^

In this contribution, we investigate whether the skin of water (the air–water interface) is so unstable that airborne microdroplets can spontaneously produce H_2_O_2_, or whether another process is occurring. We investigate the origins of approx. 1 μM H_2_O_2_ in condensed and sprayed water microdroplets *via*^1^H-NMR, to answer the following interrelated fundamental questions:

(1) Is H_2_O_2_ formation in water microdroplets influenced by the nature of the nebulizing gas (*viz.*, N_2_ or O_2_)?

(2) Would the H_2_O_2_ concentration in condensates collected in an inert gaseous environment be the same or different if the solid surface composition is varied (*e.g.*, a SiO_2_/Si wafer or stainless steel)?

(3) Is the ‘micro’ scale of droplets necessary for the spontaneous formation of H_2_O_2_ at aqueous interfaces? What would the result be if pellets of a solid material (*e.g.*, aluminum or mild steel) were immersed in bulk water, or if a film of water was sandwiched between two solid surfaces to eliminate the air–water interface from the picture?

(4) What is the role of dissolved oxygen (in water) in this chemical transformation? If the dissolved oxygen were removed from the water and sprayed, would H_2_O_2_ still form?

(5) During the spontaneous H_2_O_2_ formation at the solid–water interface, do water molecules transfer electrons to the solid and therefore reduce it?^11,15^

(6) Which aqueous interface would produce more H_2_O_2_ for a fixed area: the air–water interface or the solid–water interface (solid refers to common materials, such as glass, steel, *etc.*)?

## Results

**Fig. 1:**
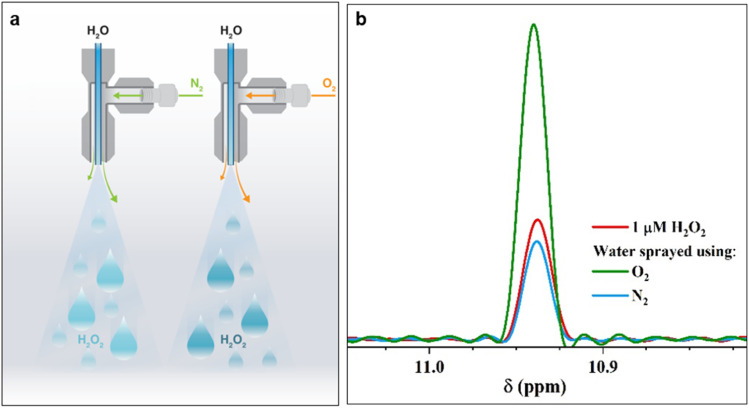
Pneumatic spraying of water into microdroplets produces H_2_O_2_. (a) Illustration of the experimental setup: as the nebulizing gas changes from N_2_(g) to O_2_(g), the H_2_O_2_(aq) concentration increases from 1.0 ± 0.2 μM to 3.0 ± 0.2 μM. (b) Representative ^1^H-NMR spectra of the reference standard, 1 μM H_2_O_2_ solution (red); ^1^H-NMR spectra of H_2_O_2_(aq) in water microdroplets nebulized by high pressure (100 psi) N_2_ (blue) and O_2_ (green) gases at an injection rate of 25 μL min^−1^. A description of our experimental set up is presented in ESI Section S1† along with photographs (Fig. S1†).

In a controlled gaseous environment (N_2_(g), unless specified otherwise) afforded by a clean glovebox, we collected water microdroplets formed *via* pneumatic sprays (Fig. S1 and Section S1 present the details†) or by condensing the vapor generated by gently heating water (60 °C) onto cold surfaces. First, we probed the effects of the nebulizing gas (N_2_ or O_2_) on the H_2_O_2_(aq) concentration. We applied the flow rates suggested by Zare & co-workers^59^ to maximize the H_2_O_2_ formation: a water flow rate of 25 μL min^−1^ through a 0.10 mm-wide silica capillary, nebulizing N_2_(g) gas at 100 psi shearing through an outer concentric tube of 0.43 mm in diameter (Fig. 1a & S1†). The spray was collected in a custom-built glass container, as described previously^48^ (Fig. S1†). The quantification of H_2_O_2_(aq) relied on ^1^H-NMR *via* the remarkable protocol of Bax *et al.*,^60^ which was also followed by Zare & co-workers.^41,59^ We employed a Bruker 950 MHz Avance Neo NMR spectrometer equipped with a 5 mm *Z*-axis gradient TCI cryoprobe at 275 K. During each measurement, a 6 ms Gaussian 90° pulse was applied to selectively excite the protons of H_2_O_2_, followed by a 53 ms acquisition time corresponding to 1024 detection points with a spectral width of 9615 Hz. Over 50 000 scans were collected with a recycle delay of 1 ms between the scans. With this technique, we observed H_2_O_2_(aq) down to approx. 50 nM detection limit (see Fig. S2† for a representative calibration plot).

The ^1^H-NMR results confirmed the presence of 1.0 ± 0.2 μM H_2_O_2_ in water microdroplets sprayed using N_2_ gas (Fig. 1). Next, when we switched the nebulizing gas to O_2_, keeping the water flow the same, the H_2_O_2_ concentration increased to 3.0 ± 0.2 μM (Fig. 1). Two crucial questions arose, which we address next: (i) If this phenomenon is driven by an ultrahigh instantaneous electric field at the air–water interface, then why does the nebulizing gas influence it; (ii) Could the solid–water interface drive this chemical transformation?

**Fig. 2:**
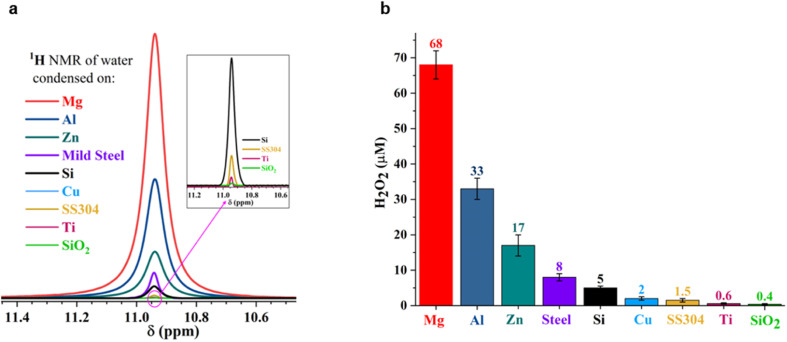
Concentration of H_2_O_2_(aq) formed in condensed water microdroplets varies with the nature of the solid substrate. (a) Selective-excitation ^1^H-NMR spectra for H_2_O_2_ quantification following the protocols developed by Bax *et al.*^60^ (b) Measured H_2_O_2_(aq) concentrations in condensates collected from the various substrates varied by over two orders of magnitude, confirmed *via*^1^H-NMR and HPAK. The air–water interfacial area of the microdroplets was not too dissimilar in these scenarios; thus, the solid–water interface drives the formation of H_2_O_2_.

To answer these questions, we compared the amount of H_2_O_2_(aq) formed in water microdroplets condensed onto a variety of smooth and flat substrates: SiO_2_/Si wafers, polished titanium, polished stainless steel (SS304), polished mild steel, silicon surfaces (obtained by the reactive ion etching of SiO_2_/Si wafers), polished copper (Cu), polished magnesium alloy (Mg alloy, AZ31B), and polished aluminum (Al) (see Methods for details). Mechanical polishing was performed using emery paper with a grit size ranging from 400 to 1500 to remove the native oxide layer. While the size distribution of the microdroplets formed on these substrates did not vary significantly because of their superhydrophilic nature, a dramatic difference in the amount of H_2_O_2_(aq) occurred in the condensates, depending on the nature of the substrate (Fig. 2). For instance, as we replaced the SiO_2_/Si wafer substrate with a Mg alloy (AZ31B), the H_2_O_2_ concentration rose from 0.4 ± 0.2 μM to 68 ± 5 μM (Fig. 2). In this four year-long investigation of this phenomenon, predominantly with null results, this was the first time we observed the formation of ppm-level H_2_O_2_ (aq) in water microdroplets in the absence of O_3_(g).

Notably, the H_2_O_2_ concentrations reported in Fig. 2 were those obtained on freshly prepared surfaces (*i.e.*, without a native oxide layer). Over time, as the condensation experiments were repeated on the same surface, the extent of the H_2_O_2_(aq) formation decreased, underscoring the importance of the solid–water interface. For instance, the H_2_O_2_ produced on a freshly prepared Al surface was around 30 to 35 μM, which decreased to 7 to 8 μM in the second cycle and to 4 to 5 μM in the third cycle (each separated by 10 min).

**Fig. 3:**
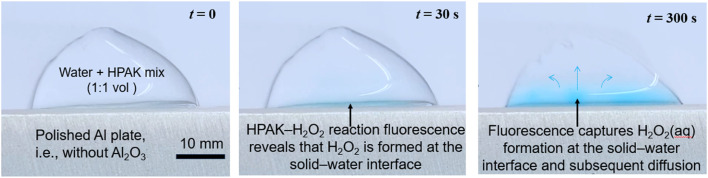
Time-dependent formation of H_2_O_2_(aq) in a macroscopic droplet of a 1 : 1 mixture of water and HPAK reaction mixture on an Al plate (see ESI Movie S1†). Within seconds, H_2_O_2_(aq) formation at the Al–water interface is evident, proving that the size of the droplet and air–water interface do not matter. The solid–water interface drives this chemical transformation. The Al surface is superhydrophilic, and water spreads on it as a film. Therefore, the Al plate was placed vertically on a polystyrene sheet and formed a 1 ml droplet resting on the Al edge. The 1 : 1 mixture of water and HPAK reaction on polystyrene did not yield the faintest blue fluorescence visible to the naked eye.

Having identified that microdroplets placed on common materials, such as (polished) aluminum, produce ppm-level H_2_O_2_, we assessed the importance of the droplet size in this chemical transformation. We formed a 1 : 1 volumetric solution of deionized (DI) water with the HPAK reaction mixture and placed a macroscopic 1000 μL droplet (a base diameter of 12 000 μm) onto an Al plate. Within a few seconds, we observed a sharp blue fluorescence – proof of the formation of H_2_O_2_(aq) – with an unambiguous gradient emanating from the Al–water interface (Fig. 3 and ESI Movie 1†). Judging by the fluorescence intensity, the local concentration of H_2_O_2_(aq) at the solid–liquid interface is at the ppm level (*i.e.*, the air–water interface produced no fluorescence visible to the naked eye).

**Fig. 4:**
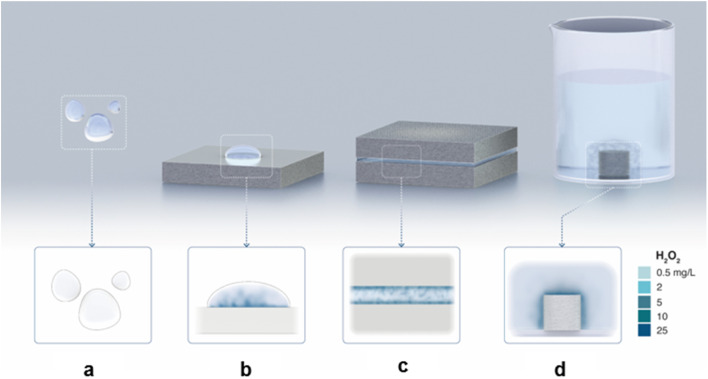
Illustration capturing the experimental observations: (a) No H_2_O_2_ is formed in the water microdroplets suspended in the air (*i.e.*, without any contact with a solid). (b) H_2_O_2_ formation in water microdroplets on a solid substrate. (c) H_2_O_2_ formation in a water film between two solids. (d) H_2_O_2_ formation in bulk water at the solid–water interface. In the latter two cases, the air–water interface was practically eliminated. See text above and Table S1† for experimental details.

Building on this experiment, we reduced the air–water interfacial area from this three-phase system by (i) layering 1 ml of fresh DI-water–HPAK 1 : 1 mixture between two 20 × 20 cm^2^ Al plates and (ii) immersing freshly polished Mg pellets (6.2 cm^2^) in 5 ml of bulk DI water–HPAK 1 : 1 mixture (Fig. 4c and d). In all these scenarios, we discovered that the solid–water interface was the site for the spontaneous H_2_O_2_ formation, and the air–water interface had a negligible effect, if any. This observation is based on the distinct color gradient at the solid–water interface. After these experiments, we collected the water samples (films or bulk) and took ^1^H-NMR measurements (Table S1†). The H_2_O_2_ concentration produced by a sessile water droplet (∼1 ml) placed on a freshly polished Al plate and the scenarios (i) and (ii) listed above after 1 minute was 1.4 ± 0.5 μM, 39 ± 6 μM, and 2.5 ± 0.6 μM, respectively. A systematic study of the effects of the solid–water surface area and the effect of time is underway. These results unambiguously establish that the spontaneous H_2_O_2_ production in water does not necessitate microscopic droplets or the air–water interface, and it can occur even in bulk water when specific solid materials are introduced.

**Fig. 5:**
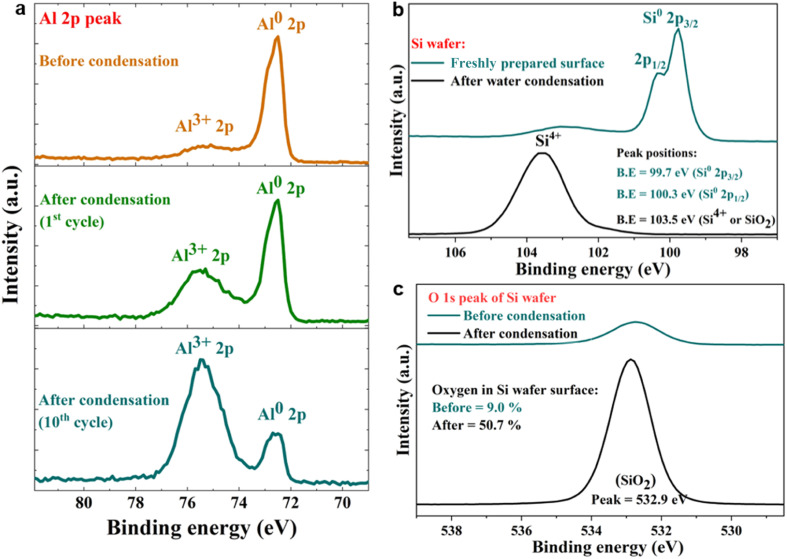
Representative XPS analysis of freshly polished metallic surfaces before and after contact with water. (a) High-resolution spectra of Al 2p before the condensation of water reveal a dominant metallic Al^0^ peak at 72.6 eV. After condensation cycles, the Al^0^ peak shrinks while the oxidized Al^3+^ peak (at 75.4 eV) increases. (b) High-resolution spectra of Si 2p before the water condensation cycle reveal a Si^0^ peak at 99.7 eV. After a water condensation cycle, the Si^0^ peak shrinks, and the Si^4+^ peak at 103.5 eV increases. (c) High-resolution XPS spectra of the O 1s peak on a freshly etched Si surface before and after a water condensation cycle. The surface oxygen composition increases from 9.0% to 50.7% after a condensation cycle due to the formation of the oxide layer.

Next, we probed the surfaces before and after contact with water *via* X-ray photoelectron spectroscopy (XPS), which revealed that the spontaneous H_2_O_2_ formation at the water-solid surface was accompanied by substrate oxidation. For instance, on contact with water (condensed or both), metallic Al (Al^0^) was oxidized to Al^3+^, and semiconductor silicon (Si^0^) was oxidized to the Si^4+^ oxidation state (Fig. 5a–c). The water microdroplets spread and merged during each cycle, covering the entire (superhydrophilic) solid surface by the end of each cycle. This finding contradicts the claims that during H_2_O_2_ formation at the solid–water interface, OH^−^ ions are oxidized to OH˙ (or H_2_O molecules are oxidized to H_2_O^+^),^11,15^ because, if this were true, the solid surface would be getting reduced, which is not the case. An in depth characterization of oxidation products is beyond the scope of this study and will be explored in the future.

**Fig. 6:**
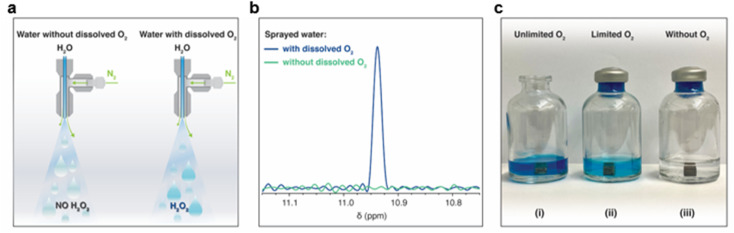
Role of the dissolved oxygen (O_2_) in water in the formation of H_2_O_2_ in microdroplets and bulk forms. (a) An illustration of the experiments in which water containing dissolved O_2_ and deoxygenated water was sprayed to form microdroplets. The microdroplets were collected in a glass vial, and H_2_O_2_(aq) was quantified. (b) Within a detection limit of 50 nM, ^1^H-NMR revealed that no H_2_O_2_ formed in the deoxygenated water, whereas H_2_O_2_(aq) was readily detected in the presence of dissolved O_2_. The air–water interface was common in both scenarios; thus, these experiments prove that the H_2_O_2_(aq) formation happens at the solid–water interface and dissolved O_2_(aq) is the limiting factor. (c) In another experiment, we prepared a 1 : 1 mixture of the HPAK reaction mixture with deoxygenated water and for the control case prepared the mixture with water containing dissolved O_2_(aq). Next, H_2_O_2_ formation in the following three scenarios was investigated: (i) an Mg pellet was added to the 1 : 1 mixture saturated with the ambient O_2_(g) and the vial was exposed to the ambient air (*i.e.*, unlimited oxygen case); (ii) an Mg pellet was added to the 1 : 1 mixture saturated with dissolved oxygen O_2_(g), and the vial was sealed (*i.e.*, the limited oxygen case); and (iii) a pellet was added to the 1 : 1 mixture without dissolved O_2_(g), and the vial was sealed (*i.e.*, the without O_2_ case). In the absence of dissolved O_2_(aq), no H_2_O_2_(aq) formed within the detection limit of 0.25 μM, whereas it appeared readily in the presence of O_2_(aq), demonstrating that this chemical transformation occurs at the solid–water interface and that dissolved O_2_(aq) is a reactant. Therefore, H_2_O_2_ formation is not a property of the air–water interface or dependent on the size of the droplets. (Scale bar: the diameter of the pellet is 1 cm).

Following the XPS study, we noticed that the H_2_O_2_ formation was accompanied by surface oxidation. Therefore, we got curious whether it was due to the reduction of the dissolved oxygen.^61^ To examine this, we first removed dissolved O_2_ from the water by heating it in an autoclave to its boiling point, followed by N_2_(g) bubbling for 45 minutes and then sealing it inside an N_2_-purged container (methods). This treatment reduced the O_2_(aq) concentration to <0.01 mg L^−1^. Microdroplets of O_2_-free water were formed *via* pneumatic spraying using N_2_(g) in an N_2_ environment and collected in glass containers (following the same protocol). The H_2_O_2_(aq) concentration was compared with that in the microdroplets formed with water containing dissolved O_2_(g) (Fig. 6a). Remarkably, in the absence of O_2_(aq), no H_2_O_2_(aq) was observed *via* 1H-NMR (detection limit ≥50 nM; Fig. 6b). Next, we tested the effects of dissolved O_2_(g) on the formation of H_2_O_2_(aq) in bulk water by adding metallic pellets (Mg or Al). We tested the following three scenarios:

(i) An Mg pellet was added to water saturated with the ambient O_2_(g), and the vial was left open in an O_3_-free ambient environment.

(ii) An Mg pellet was added to water saturated with ambient oxygen O_2_(g) and then sealed.

(iii) An Mg pellet was added to O_2_-free water in an N_2_(g) environment and sealed.

The vial open to the ambient air had significantly higher H_2_O_2_(aq) than that in the sealed vial containing water saturated with dissolved O_2_(g). Thus, the formation of H_2_O_2_ at the solid–water interface consumes dissolved O_2_(g) (*i.e.*, it is the limiting factor). We also characterized the consumption of the dissolved O_2_(g) before and after adding the pellets and found that it decreased over time (Fig. S4†). Notably, in the absence of dissolved O_2_(g), we did not observe H_2_O_2_(aq) within the detection limit of 50 nM (Fig. 6b). These results unambiguously establish (i) the importance of dissolved O_2_(g) in this chemical transformation and that (ii) the air–water interface of microdroplets is incapable of forming H_2_O_2_.

## Discussion

**Fig. 7:**
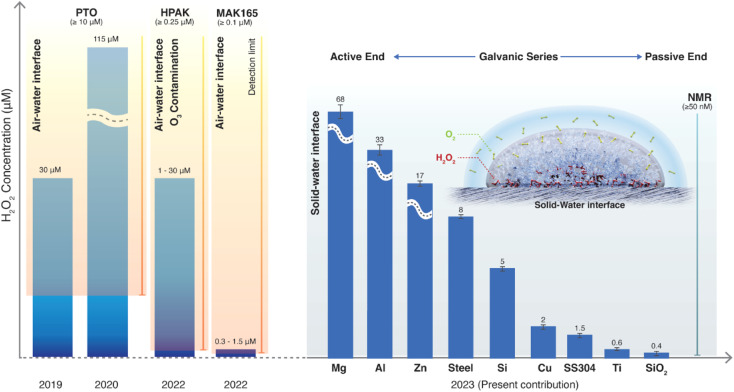
Current understanding of the spontaneous H_2_O_2_ formation in water microdroplets since its first report in 2019. Initial papers by Zare & coworkers used the PTO assay (detection limit ≥10 μM) and reported 30 and 110 μM H_2_O_2_(aq) in sprays and condensates, respectively.^9,10^ In 2022, it was revealed that ambient O_3_(g) could cause severe artifacts in these microdroplet experiments using HPAK (detection limit ≥0.25 μM).^48^ In 2023, using ^1^H-NMR (detection limit ≥0.04 μM), it was contended that in an O_3_-free environment, the air–water interface still produces H_2_O_2_ (∼1 μM).^59^ In the present contribution using ^1^H-NMR, we reveal that the solid–water interface is the site for H_2_O_2_(aq) formation, and the air–water interface does not contribute to H_2_O_2_ formation (quantified within the detection limit of ≥0.05 μM). If dissolved O_2_ is removed from the water, H_2_O_2_(aq) is not observed within the detection limit. Next, depending on the nature of the substrate that water contacts (as microdroplets or a film or as bulk water), the amount of H_2_O_2_(aq) formed follows the classic Galvanic series.^62^

We draw together the results of this study and previous scientific reports to discuss the mechanisms underlying the formation of H_2_O_2_ in interfacial water (Fig. 7). The first finding is that the amount of H_2_O_2_(aq) formed in water condensates (or sprayed microdroplets) depends only on the nature of the surface on which it is collected (Fig. 2 and 3). In other words, the air–water interface or the size of the microdroplets has no bearing on the H_2_O_2_(aq) formation (Fig. 3, 4d, and S5†). For instance, as the air–water interface is reduced (or eliminated) from the picture, *via* layering water films between solid plates (Fig. 4c) (or by introducing solid pellets into bulk water) the formation of H_2_O_2_(aq) remains unaffected (Fig. 4c, d and S5†). The second crucial finding is that if the dissolved O_2_ is removed from the water, there is no evidence for H_2_O_2_(aq) formation within the detection limit (Fig. 6a–c). This observation contradicts the suggested mechanism for H_2_O_2_(aq) formation due to the charge transfer between positively (H_3_O^+^ rich) and negatively charged (OH^−^ rich) microdroplets.^42,46^

Next, the XPS results demonstrate that the formation of H_2_O_2_(aq) is accompanied by the oxidation of the solid surface and the reduction of dissolved O_2_ (Fig. 5). Fig. S4† illustrates how the absolute concentration of O_2_(aq) decreases during H_2_O_2_ formation. When we evaluated the various commercially available materials used in this study in terms of their ability to form H_2_O_2_(aq) in (air-equilibrated) water, the trend followed the Galvanic series: Mg > Al > Zn > mild steel > Si > Cu > stainless steel (SS304) > Ti > SiO_2_/Si wafer (Fig. 7). Therefore, these findings refute the previous speculations regarding the oxidation of OH^−^ ions to OH˙ (or the oxidation of H_2_O to H_2_O^+^) and the reduction of the solid surface during the H_2_O_2_ formation at the solid–water interface.^11,15^

We postulate that the initiation of this chemistry involves the reduction of dissolved O_2_(aq) by the solid surface; that is, the surface transfers two electrons into interfacial O_2_(aq), transforming it into a highly reactive peroxide dianion (O_2_˙^2−^).^63^ We anticipate this anion species to hover near the solid–water interface due to electrostatic attraction. Next, the anion reacts with interfacial water molecules to form H_2_O_2_ and hydroxide ions^61,63^ (the reactions below capture this logic):

1 O_2_ + 2e^−^ → O_2_˙^2−^

2 O_2_˙^2−^ + 2H_2_O → H_2_O_2_ + 2OH^−^

Reaction (2) indicates that the H_2_O_2_(aq) formation is accompanied by pH enhancement. The pH of the condensate collected on the Mg plate was around 7.9 ± 0.2, whereas the pH of the water reservoir used for gentle heating was 5.6 ± 0.1. The H_2_O_2_(aq) formation rate is the highest when the surface is free of native oxide and slows down as the oxide layer grows, which was also noticed in our experiments. Similar reaction schemes have been proposed recently, and in some of them, O_2_ is the byproduct.^40^ If this were true, then the H_2_O_2_(aq) formation due to the addition of a metal pellet (Mg or Al) to bulk water would be the same whether the dissolved O_2_ content was (i) unlimited (Fig. 6ci), (ii) limited (Fig. 6cii) or (ii) nil (Fig. 6ciii), but that is not the case. An in-depth investigation of the reaction intermediates (Reactions (1) and (2)) and the contribution of metal pellets on water-splitting reactions and the water pH is underway.

## Conclusion

These findings put to rest several myths surrounding the spontaneous formation of H_2_O_2_ at the air–water interface, including the instantaneous ultrahigh electric fields, the “microscale” of droplets,^9,10,12–14,40,41,44,45,59^ and arguments based on charge transfer between microdroplets.^42,46^ For water containing dissolved O_2_, which is commonplace in environmental and applied scenarios, the solid–water interface is the site where O_2_(aq) reduces and forms H_2_O_2_(aq). One can therefore expect trace level (<0.5 μM) H_2_O_2_ to be produced at (clean) glass–water interfaces in laboratories routinely that, depending on the surface-to-volume ratio, may impact ultrasensitive investigation of aqueous interfaces and engineering processes such as semiconductor device fabrication. Notably, the ability of a solid to drive this chemical transformation depends on its position in the Galvanic series. For example, Mg and Al have low oxidation resistance; therefore, they form higher H_2_O_2_(aq), and, in contrast, Ti and stainless steel have high oxidation resistance, forming a lower amount of H_2_O_2_(aq). Next, our XPS experiments have revealed that the solid surface gets oxidized during the formation of H_2_O_2_, which refutes the suggestion that during the formation of H_2_O_2_ at the solid–water interface, water molecules transfer electrons to the solid, reducing it.^11,15^

Crucially, in the absence of dissolved O_2_ in water, H_2_O_2_(aq) was not observed in pneumatic sprays or in bulk water containing pellets of Mg or Al (down to the 50 nM detection limit). This demonstrates that (i) the air–water interface of sprayed microdroplets and the putative (instantaneous) ultrahigh electric field therein are not capable of spontaneously forming H_2_O_2_ (Fig. 6), and (ii) the presence of dissolved O_2_ is a required condition for the solid–water interface to form H_2_O_2_. Therefore, we submit that the latest claims^59,64^ of the formation of ≤3 μM H_2_O_2_(aq) in water microdroplets (containing dissolved O_2_) suffered from artifacts arising due to the unavoidable physical contact of water with solid surfaces (*e.g.*, during sample preparation, collection, and analysis), and evaporative concentration.^48^

When water microdroplets were formed by nebulizing with O_2_(g), it increased the O_2_(aq) concentration, promoting the formation of H_2_O_2_(aq) at the solid–water interface. Conversely, if the water is devoid of O_2_(g), H_2_O_2_ is not formed spontaneously at the solid–water interface. In that scenario, the air–water interface can contribute to the H_2_O_2_ formation in the following two ways: (i) transfer O_2_(g) to be reduced at the solid–water interface to form H_2_O_2_(aq) (see the inset in Fig. 7); or (ii) transfer O_3_(g) to oxidize water to form H_2_O_2_(aq) without the necessity of the solid–water interface. We hope these findings will advance the current knowledge of aquatic chemistry and prove relevant to corrosion science, electrochemistry, and soil chemistry.

## Materials and methods

### Chemicals

Deionized water was obtained from a Milli-Q Advantage 10 set-up (18.2 MΩ cm resistivity). This study used commercially available 30% hydrogen peroxide (H_2_O_2_) solution (Sigma-Aldrich CAS no. 7722-84-1) and deuterium oxide ((D_2_O, Catalog no. 3000007892).

### Spraying microdroplets

Inside a glove box with a controlled N_2_(g) atmosphere to prevent ambient contamination, the water was injected using a stainless steel capillary tube with an inner diameter of 100 μm using a syringe pump (PHD Ultra, Harvard Apparatus). Ultra-pure N_2_/O_2_ was pushed through a coaxial stainless steel sheath with an inner diameter of 430 μm to nebulize the water stream (Fig. S1†). The liquid water flow rate was 25 μL min^−1^, and approximately 2 ml of microdroplet volume was collected in clean glass vials for further analysis.

### Deoxygenation of water

Water was heated in an autoclave to its boiling point, followed by cooling *via* N_2_(g) bubbling for 45 min, lowering the temperature to about 40 °C. An O_2_ sensor (WTW Multi 3320) measured the dissolved O_2_ concentration in water with a detection limit of 0.01 mg L^−1^. After this treatment, we could not observe a signal for the O_2_(aq), meaning it was below the detection limit. Next, the water was quickly transferred to a glove box filled with N_2_ gas, where glass bottles were filled and sealed. The water sealed in an N_2_(g) filled glove box was autoclaved at 121 °C for 10 min to remove organic contamination on the vials.

### Substrates for condensation

Silicon (SiO_2_/Si) wafers of about 300 μm thickness, 10 cm in diameter, and 2 μm-thick thermally grown oxide were purchased from Silicon Valley Microelectronics (Catalog #SV010, p-type and 100 orientation). Fresh Si surfaces were prepared by etching the SiO_2_ layer *via* reactive ion etching (using C_4_F_8_ and O_2_(g) for 5 mi) inside the KAUST cleanroom.^65^ Directly afterward, condensation experiments were performed on the etched surfaces, and water samples were collected in clean glass vials for ^1^H-NMR analysis. Next, the following commercially available plates comprising metals or metallic alloys were used: Mg alloy (AZ31B, Thermo Scientific, Catalog No. AA14066RF), Al (Fisher Scientific, Catalog no. AA42124RF), mild steel, stainless steel (SS304), Zn (Thermo Scientific, Catalog No. AA11914FI), Cu (Thermo Scientific Chemicals, Catalog no. AA43822KS), and Ti (ASTM B 265 Trinity Brand Industries INC part #6T-5). The native oxide on the metal plates was removed *via* mechanical polishing using SiC emery papers with a grit size of 400 to 1500, followed by cleaning with pressurized N_2_ gas. For the condensation experiments, DI water was heated to 60 °C inside a closed chamber to produce the water vapor. Water microdroplets formed on cooled substrates (placed directly on ice) were collected using a low-pressure N_2_ gas stream and transferred to NMR tubes and glass vials for further analysis.

### Hydrogen peroxide assay kit (HPAK) assay

The H_2_O_2_ concentration of the condensed water was quantified using the hydrogen peroxide assay kit (Fluorometric-Near Infrared, Catalog # ab138886). It contains a unique AbIR peroxidase indicator that produces fluorescence independent of the solution pH in the range of 4 to 10. Its maximum excitation wavelength is 647 nm, and the maximum emission is 674 nm. The horseradish peroxidase enzyme catalyzes the reaction between H_2_O_2_ and the indicator and enhances the fluorescence signal. This action facilitates the linear detection range from 30 nM to 10 μM. The calibration curve (Fig. S3†) was realized by adding 50 μL of an H_2_O_2_ standard solution from a concentration of 50 nM to 10 μM into 50 μL of the H_2_O_2_ reaction mixture using a black 96-well microtiter-plate, and the SpectraMax M3 microplate reader (Molecular Devices LLC). The analysis software was SoftMax Pro 7. The water microdroplets were analyzed similarly by mixing 50 μL of each sample with the H_2_O_2_ reaction mixture, obtaining the respective concentration from the calibration curve.

### NMR spectroscopy analysis and sample preparation

No chemical was added to adjust the pH of the samples to avoid contamination. In each case, 10 μL of D_2_O was added to 490 μL analyte in regular 5 mm quartz NMR tubes for testing. All NMR measurements were conducted on a Bruker 950 MHz Avance Neo NMR spectrometer equipped with a 5 mm *Z*-axis gradient TCI cryoprobe at the temperature of 275 K. During the measurement, a 6 ms Gaussian 90° pulse was applied to selectively excite the protons of H_2_O_2_, followed by a 53 ms acquisition corresponding to 1024 detecting points with a spectral width of 9615 Hz. Over 50 000 scans were collected with a recycle delay of 1 ms between scans. The NMR data were analyzed using TopSpin 4.2.0 software.

### XPS measurements

A Kratos Axis Supra instrument equipped with a monochromatic Al Kα X-ray source (*hν* = 1486.6 eV) operating at a power of 75 W under UHV conditions in the range of 10^−9^ mbar was used to obtain the data. All spectra were recorded in hybrid mode using magnetic and electrostatic lenses and an aperture slot of 300 × 700 μm. The high-resolution spectra were acquired at a fixed analyzer pass energy of 20 eV. The adventitious carbon (C 1s) peak at 284.5 eV was used as a reference for calibrating all peaks.

## Data availability

All data needed to evaluate the conclusions in the paper are present in the paper or the ESI.†

## Author contributions

HM conceived the research plan and oversaw its execution. ME designed and performed the condensation and spray experiments inside the glovebox and collected the ^1^H-NMR and HPAK data. ME and HM analyzed the data and wrote the manuscript together.

## Conflicts of interest

The authors declare no competing interests.

## Supplementary Material

SC-015-D3SC06534K-s001

SC-015-D3SC06534K-s002
