# Supplementary material for: Busting the myth of spontaneous formation of H2O2 at the air–water interface: contributions of the liquid–solid interface and dissolved oxygen exposed
Source: Chem Sci. 2024 Jan 24;15(9):3093–103. doi: 10.1039/d3sc06534k (PMC10901496; doi:10.1039/d3sc06534k)
Supplement: SC-015-D3SC06534K-s002 [file SC-015-D3SC06534K-s002.pdf]

Electronic Supplementary Material (ESI) for Chemical Science.
This journal is © The Royal Society of Chemistry 2024

Supplementary Information

Busting the Myth of Spontaneous Formation of H_2O_2 at the Air–Water Interface: Contributions of the Liquid–Solid Interface and Dissolved Oxygen Exposed

Muzzamil Ahmad Eatoo & Himanshu Mishra*

Environmental Science and Engineering (EnSE) Program,

Biological and Environmental Science and Engineering (BESE) Division,

King Abdullah University of Science and Technology (KAUST),

Thuwal, 23955-6900, Kingdom of Saudi Arabia

Water Desalination and Reuse Center (WDRC),

King Abdullah University of Science and Technology (KAUST), Thuwal, 23955-6900, Kingdom of Saudi Arabia

Center for Desert Agriculture (CDA),

King Abdullah University of Science and Technology (KAUST), Thuwal, 23955-6900, Kingdom of Saudi Arabia

*Himanshu.Mishra@kaust.edu.sa

Section S1: Experimental setup

Water microdroplet generation via sprays

We adapted the experimental setup built by Gallo et al.[1] to produce water microdroplets. In a coaxial system, water was injected through an inner tube with a 100-µm diameter using a syringe pump (PHD Ultra, Harvard Apparatus). Dry N_2(g) was pushed through the outer tube with a 430-µm diameter. Additionally, HPLC-grade water was used, and a glass cell (equipped with a tiny opening to prevent pressure build-up) was employed to collect microdroplets while minimizing ambient contamination. The water flow rate was set to 25 µL/min, whereas the gas (N_2 or O_2) pressure was 100 psi.

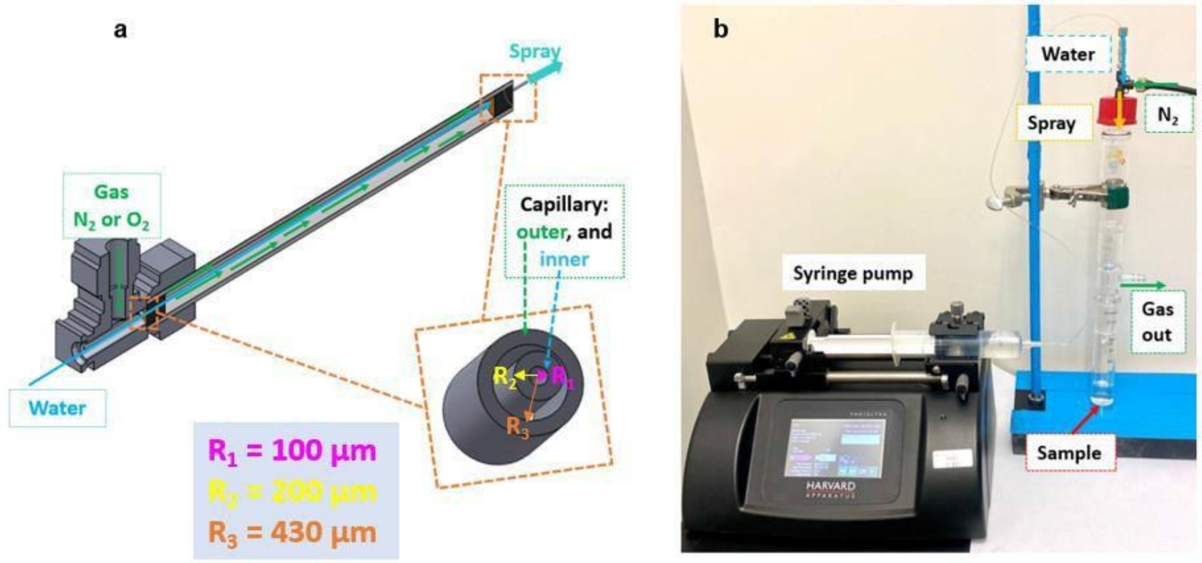

Fig. S1: **a)** Schematics of pneumatic spray setup. **b)** Photograph of spray setup connected to a removable glass flask for sample collection, comprising two coaxial capillary tubes. Liquid flows through the inner tube, whereas nitrogen gas flows through the outer annulus. Ultrapure water samples were injected via a syringe pump, and a high-pressure cylinder supplied N_2/O_2 gas. Sprayed microdroplets were collected in a clean glass flask until a sufficient analyte volume was collected.

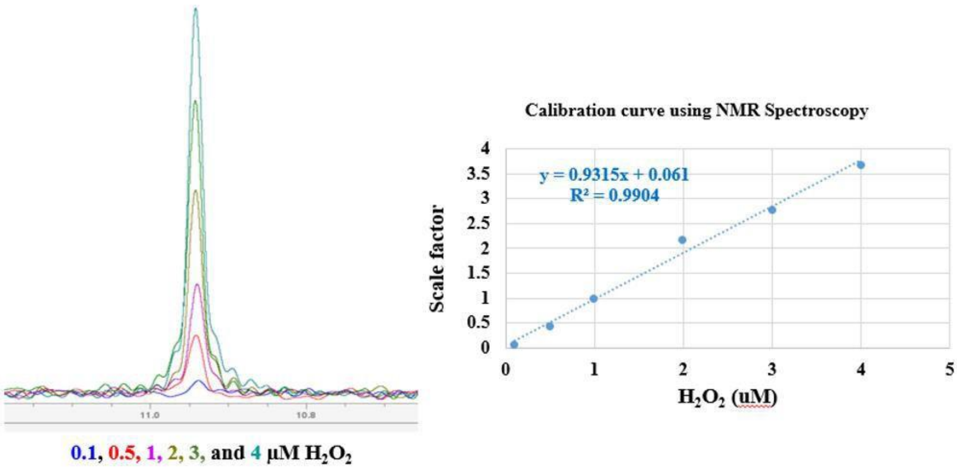

Fig. S2: Quantification of H_2O_2 by ^{1}H-NMR spectroscopy. Standard samples of known H_2O_2(aq) concentrations were prepared by diluting a concentrated 30% (v/v) stock solution using HPLC-grade deionized water. All ^{1}H-NMR experiments were conducted at 2℃. TopSpin 4.2.0 software processed the data.

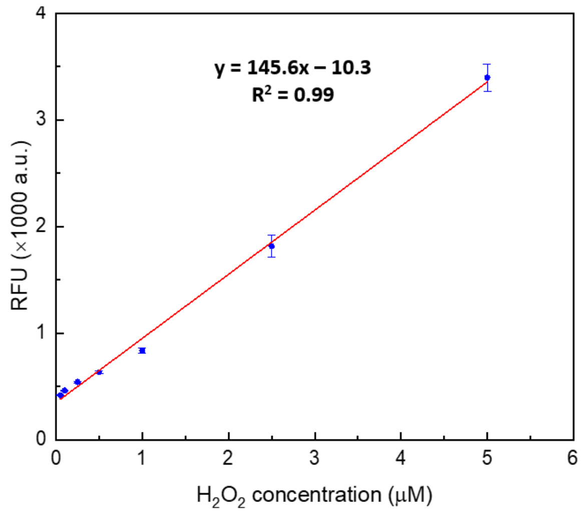

Fig. S3: Representative calibration curve for H_2O_2 measurement using the hydrogen peroxide assay kit (HPAK).

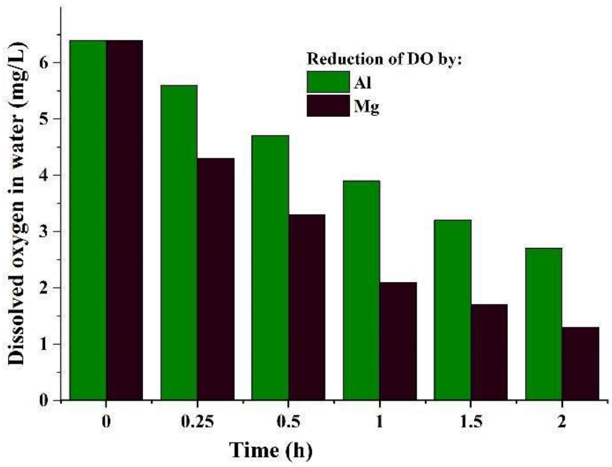

Fig. S4. Reduction of dissolved oxygen (O_2) concentration in water during H_2O_2 formation at the water–solid interface. In a typical experiment, an Al or Mg pellet was immersed in 5 mL of water in a closed vial. The dissolved O_2 concentration was measured over time using a probe (WTW Multi 3320 device) with a detection limit of 0.01 mg/L. The results revealed that the dissolved O_2 concentration reduced over time as H_2O_2 formed. Moreover, the reduction was faster with the Mg than with the Al pellet in accordance with their ability to produce $H_2O_2(aq)$.

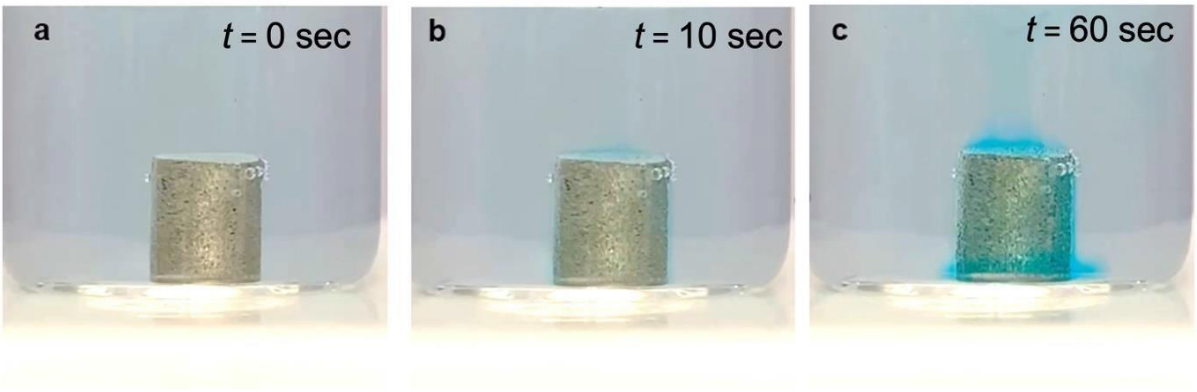

Fig. S5. An Mg pellet is immersed in a 1:1 mixture of water and HPAK, producing H_2O_2(aq) evidenced by the blue fluorescence over time, proving that this chemical transformation does not depend on the air–water interface, “microscale” water droplets, or ultrahigh (instantaneous) electrical fields.

Table S1: H_2O_2 formation in bulk water as characterized by NMR spectroscopy

Solid–water system<br>Water obtained from the Milli-Q system		Measured H_2O_2(aq) concentration after 1 min<br>(NMR spectroscopy)
1	1 mL sessile water droplet placed on a freshly polished Al plate (see Supplementary Movie S1)	1.4 ± 0.5 μM
2	1 mL water film between two freshly polished Al plates of size 200× 200 mm	39 ± 6 μM
3	A cylindrical Mg pellet with a diameter of 1 cm and height of 1.5 cm (6.2 cm^2) fully immersed in 5 mL of water	2.5 ± 0.6 μM

References

1. A. Gallo Jr, N. H. Musskopf, X. Liu, Z. Yang, J. Petry, P. Zhang, S. Thoroddsen, H. Im and H. Mishra, On the formation of hydrogen peroxide in water microdroplets, *Chemical Science*, 2022, **13**, 2574-2583.
